# Intraindividual Comparison of the Anabolic Steroid Hormone Profile Between Hormonal Contraceptive Use and the Phases of a Natural Menstrual Cycle in a Recreational Athlete: A Single‐Case Observational Study

**DOI:** 10.1155/crie/9648747

**Published:** 2026-04-08

**Authors:** Jana Nolte, Sven C. Voss, Annekathrin Martina Keiler, Kirsten Legerlotz, Petra Platen

**Affiliations:** ^1^ Department of Sports Medicine and Sports Nutrition, Ruhr University Bochum, Bochum, Germany, ruhr-uni-bochum.de; ^2^ Institute of Doping Analysis and Sports Biochemistry Dresden (IDAS), Kreischa, Germany; ^3^ Environmental Monitoring and Endocrinology, TU Dresden, Dresden, Germany, tu-dresden.de; ^4^ Department of Movement and Training Sciences, University of Wuppertal, Wuppertal, Germany, uni-wuppertal.de

**Keywords:** androgen metabolism, androstenedione, athletic performance, case report, female athletes, hormone fluctuations, testosterone

## Abstract

Endogenous androgens, including testosterone and the androgen precursor androstenedione, are known to influence muscular adaptation. However, variations in these hormones throughout the natural menstrual cycle (MC) and during oral hormonal contraception (HC) remain underexplored in the athletic population with cycle‐based training approaches. This study investigates variations in steroid hormone profiles under HC and during a natural MC in one recreational athlete. An observational study was conducted on a 25‐year‐old female recreational athlete (167 cm, 60 kg, 6 h training/exercise activity per week). Testosterone and androstenedione concentrations were measured in her urine and plasma every two to 3 days during 3 weeks of HC use (hormonal vaginal ring) and the 1‐week HC break and 11 months later during one natural MC. Hormone analysis was conducted using gas chromatography coupled with mass spectrometry for urine steroids and liquid chromatography coupled with mass spectrometry for plasma steroids. Throughout the MC, urinary total androgen and plasma androstenedione levels peaked around ovulation, followed by a decline in the luteal phase, suggesting phase‐dependent variation in androgen precursor availability. Meanwhile, plasma testosterone remained unchanged throughout the cycle. HC use resulted in lower androgen concentrations with no notable fluctuations in plasma or urine. The suppression of naturally secreted androgens and their respective fluctuations throughout MC induced by HC may have implications for training adaptations and athletic performance. Furthermore, it is essential for female athletes and their coaches to consider the use of HC in order to optimize training strategies.

## 1. Introduction

The impact of endogenous androgens, such as testosterone, and of androgen precursors, including androstenedione and other metabolites, on physiological adaptations and performance has been widely investigated, particularly in men. These hormones significantly contribute to the observed differences in performance between the sexes [1], which highlights the complexities surrounding female athletes with hyperandrogenic hormonal profiles, such as transgender women [2]. However, the fluctuations of these hormones, which are secreted by both the ovaries and the adrenal cortex [3] in women throughout the menstrual cycle (MC), as well as the influence of hormonal contraception (HC), are the subject of ongoing research and debate.

It has long been known that the MC leads to a secretion of ovarian androgens in a cycle phase‐dependent manner [4]. The first studies demonstrating cycle‐dependent testosterone and androstenedione secretion were published between 1972 and 1986 [5–7]. More recent studies emphasize that these fluctuations are often associated with a mid‐cycle peak around the time of ovulation [8–10]. The growing interest in MC‐based training approaches underscores the need for detailed physiological characterization of hormonal fluctuations before evidence‐based training recommendations can be formulated [11–13]. These findings are particularly relevant given that testosterone is a well‐established modulator of motivation and performance, enhancing both factors under certain physiological conditions [14].

HCs generally suppress the ovarian production of endogenous steroid sex hormones, including estradiol, progesterone, testosterone, and androstenedione, as well as their metabolites [15–18]. Therefore, a more precise understanding of how HCs affect physiological hormone profiles could help to inform decisions about their use in sport and identify potential risks and benefits [15–17].

In general, testosterone and androstenedione are synthesized from cholesterol, which is converted into steroid hormones via pregnenolone. In women, androgen production is regulated by both the hypothalamic–pituitary–gonadal (HPG) axis and the hypothalamic–pituitary–adrenal (HPA) axis. While ovarian androgen synthesis is primarily controlled by the gonadotropin‐releasing hormone (GnRH) and the dependent secretion of luteinizing hormone (LH) and follicle‐stimulating hormone (FSH), the adrenal cortex substantially contributes to circulating androstenedione and, to a lesser extent, testosterone via corticotropin‐releasing hormone (CRH) and subsequent adrenocorticotropic hormone (ACTH)‐mediated regulation [19, 20]. Once synthesized, steroid hormones enter the bloodstream, where they can be measured in plasma or serum. The fluctuations in blood concentrations of steroid hormones are influenced by circadian rhythms in secretion, as well as by acute physical or mental stress. In contrast to the hormonal fluctuations observed in plasma or serum, urinary concentrations of steroid metabolites (mainly glucuronides and sulfate conjugates) reflect integrated androgen metabolism over time and provide a complementary view of androgen secretion when appropriately normalized [19, 21], offering a more comprehensive view of these processes over time [20].

The aim of this single‐case observational study was to descriptively assess intraindividual variation in urinary and plasma androgen profiles during hormonal contraceptive use and across the phases of a natural MC within one recreational athlete.

## 2. Case Presentation

### 2.1. Participant

A recreational female athlete (25 years, 167 cm, 60 kg, 6 h training/exercise activity per week) participated in this case study on a voluntary basis.

During the study period, the participant voluntarily discontinued her use of HC (GinoRing 0.120 mg/0.015 mg per 24 h, active ingredients: 11 mg etonogestrel, 3.47 mg ethinyl estradiol), which allowed for the reestablishment of a natural MC. The study was approved by the institutional ethics committee of the Faculty of Sport Science at Ruhr‐University Bochum (EKS V 06/2022). All procedures were conducted in accordance with the Declaration of Helsinki, ensuring the participant’s well‐being and data protection throughout the study. The participant had provided informed consent prior to her involvement.

### 2.2. Study Design

The athlete’s hormonal profile was monitored over a period of 3 weeks during the use of HC (“use”), preceded and followed by 3 days of monitoring during the HC break (“free”), after which HC use was discontinued. Following an 11‐months wash out period, a 27‐day natural MC was observed. The MC length of 27 days was defined retrospectively based on the observed onset of menstruation. The natural MC was divided into different phases: “M”—menstruation (days of bleeding), “FP”—follicular phase (days between bleeding and ovulation), “O”—ovulation, and “LP”—luteal phase (days between ovulation and onset of bleeding).

The hormonal concentrations measured included testosterone, 5α‐androstanediol, 5β‐androstanediol, androsterone, epitestosterone and etiocholanolone in urine, and androstenedione, and testosterone in plasma. Samples were collected at two‐ to 3‐day intervals, with the exception of HC in urine, which was collected on a daily basis. 50 mL of urine was collected as morning urine (after the longest period of sleep during the night). A total of 250 μL of capillary blood was collected from the earlobe into a MAP microtube (K2 EDTA) for the analysis of plasma. The sample was collected during the morning period, specifically between 07:00 a.m. and 12:00 a.m. In the HC period, sampling commenced on day four of the hormone‐free phase and concluded on day 28 following the removal of the hormone ring after a period of 3 days. The measurement of the natural MC was initiated on day 1, which corresponded to the first day of bleeding, 11 months later, and concluded on day 27. Alcohol intake was documented and measured for the MC cycle. The results of the study are presented in the Supporting Information.

For the purpose of ovulation detection, the basal body temperature was measured via continuous 24‐h intravaginal body temperature measurement (OvulaRing, VivoSensMedical GmbH, Leipzig, Germany) and confirmed by measuring progesterone levels in plasma.

### 2.3. Sample Analysis

Urinary steroid hormones were analyzed by gas chromatography‐coupled mass spectrometry (GC‐MS/MS) [22]. Specific gravity was measured using a digital refractometer, and urinary steroid concentrations were adjusted accordingly to minimize the influence of hydration status using validated WADA procedures (according to the WADA Technical Document—TD2022DL). The plasma steroid hormones were analyzed by liquid chromatography with mass spectrometry coupling (LC‐MS/MS) [20, 23].

### 2.4. Data Analysis

Single hormonal levels in urine and plasma were plotted to visualize differences across phases for both HC and MC. A comprehensive examination of descriptive statistics and qualitative patterns was conducted to elucidate hormonal fluctuations. The plots were generated using the statistical programming language R (RStudio, University of Auckland, New Zealand). The Wilcoxon signed‐rank test was performed to compare total hormone concentrations between HC and MC.

## 3. Results

The hormonal profile of the female athlete demonstrates a distinct peak in the urine androgen hormone concentration around the time of ovulation, followed by a decline in the luteal phase (Figure 1). Androgen levels remain relatively stable and at significantly lower levels, particularly those of testosterone, when using HC. Plasma testosterone concentrations demonstrated moderate fluctuations across the MC, within a range that is consistent with expected physiological and analytical variation. Conversely, the use of HC was found to be associated with relatively stable and lower plasma testosterone levels. Plasma concentrations of androstenedione exhibited a transient increase around the ovulatory phase, followed by a decline during the luteal phase. Conversely, the use of HC resulted in consistently lower and more stable androstenedione levels.

Figure 1Course of the concentrations. (a) testosterone (ng/mL) in urine, (b) androsterone (ng/mL) in urine, (c) 5α‐androstanediol (ng/mL) in urine, (d) 5β‐androstanediol (ng/mL) in urine, (e) etiocholanolone (ng/mL) in urine, (f) epitestosterone (ng/mL) in urine, (g) testosterone (ng/mL) in plasma, and (h) androstenedione (ng/mL) in plasma during a natural menstrual cycle (colored lines) compared to a cycle under hormonal contraception (gray line). The phases of the natural cycle are color coded: M, menstruation (red); FP, follicular phase (orange); O, ovulation (purple); LP, luteal phase (green). The phases of HC are gray coded: free = HC free phase (light gray, use = HC use phase (dark gray).

**Figure:**
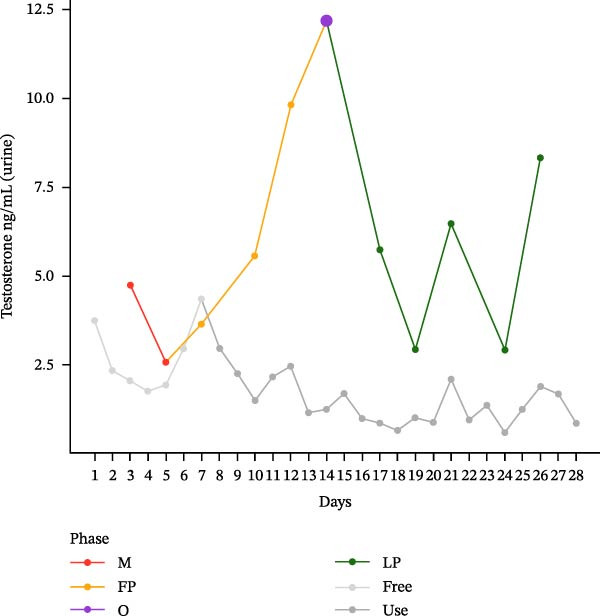
(a)

**Figure:**
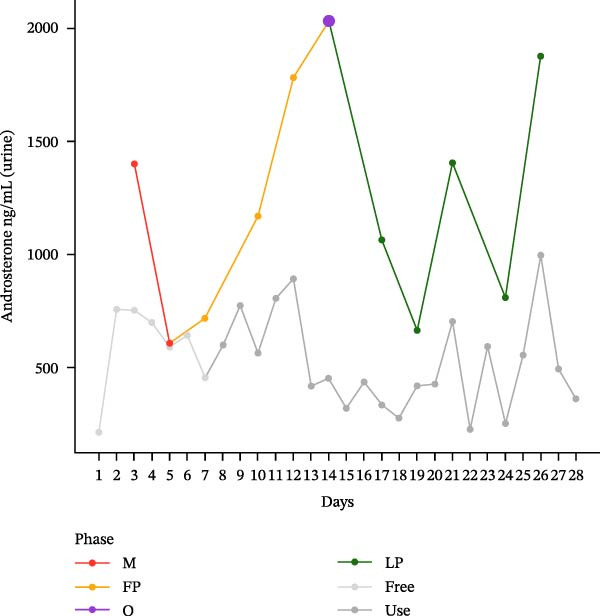
(b)

**Figure:**
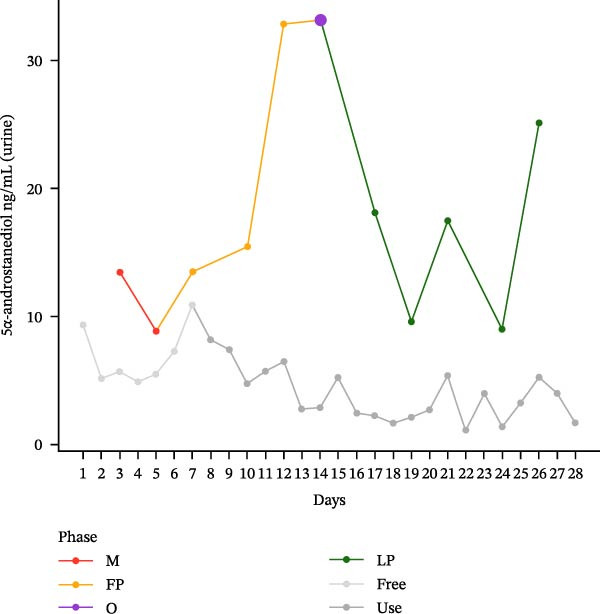
(c)

**Figure:**
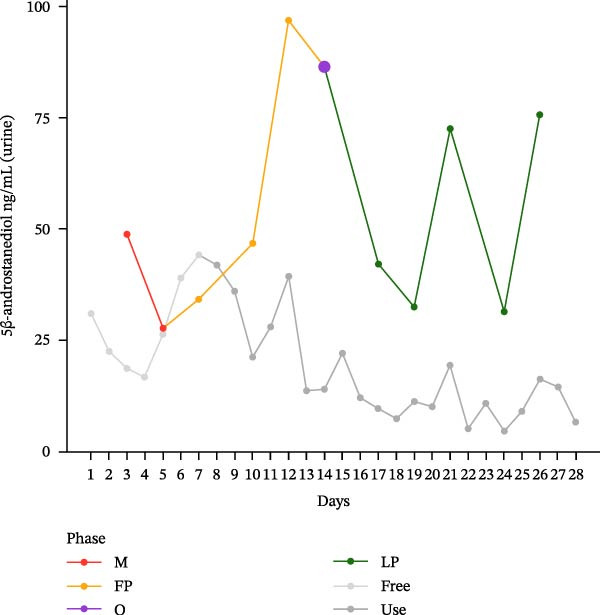
(d)

**Figure:**
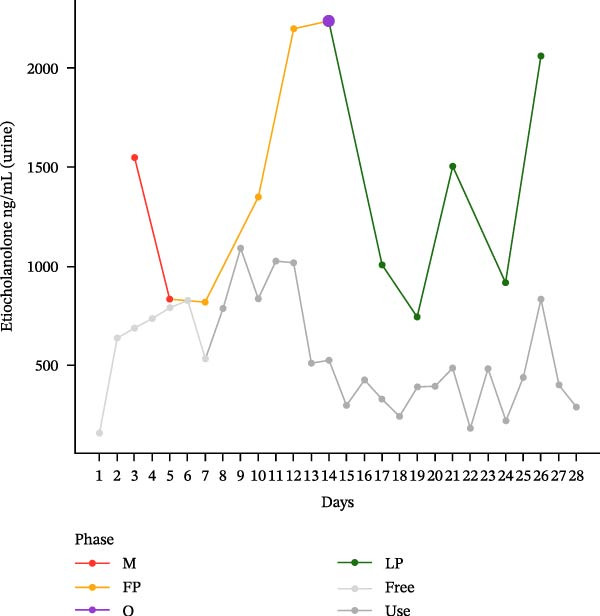
(e)

**Figure:**
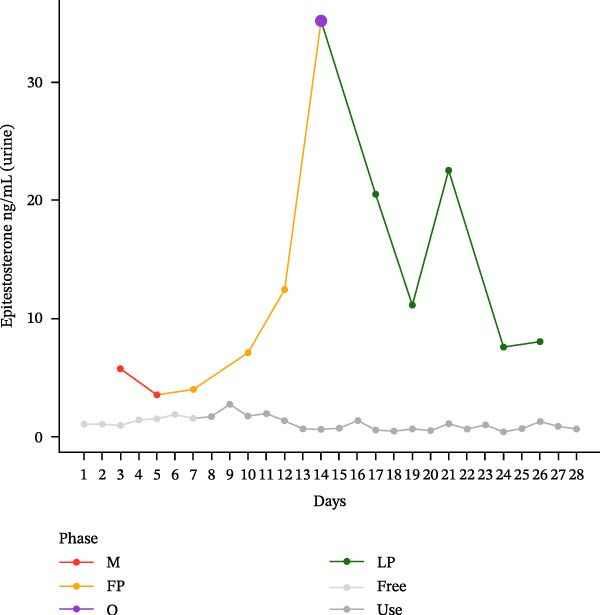
(f)

**Figure:**
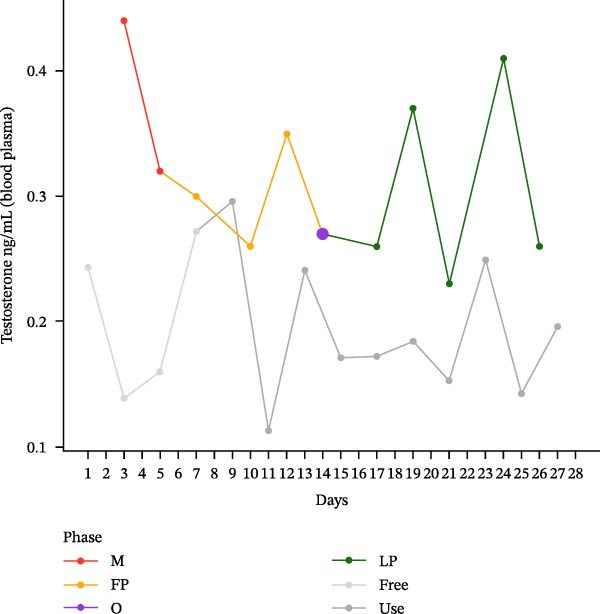
(g)

**Figure:**
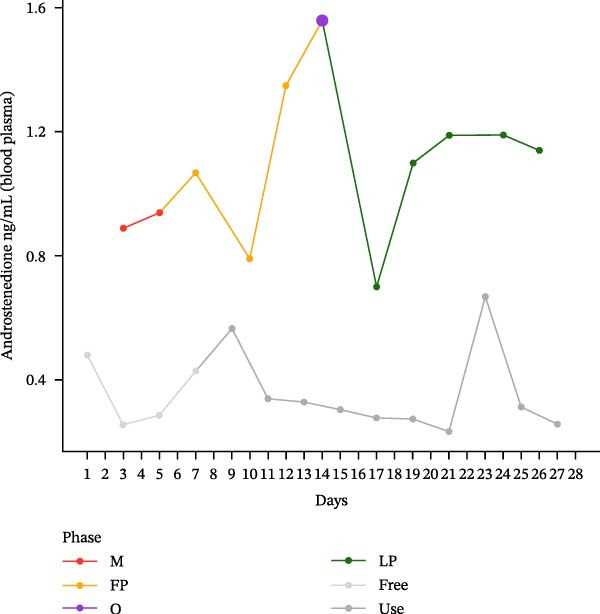
(h)

Hormone concentrations were significantly lower during hormonal contraceptive use compared to the natural MC for all seven hormone profiles (Wilcoxon signed‐rank test, *p* < 0.05) (Table 1). It appears that there are no clear differences between the HC free and the HC use phase, except for the urinary testosterone concentration. In contrast, concentrations vary between menstruation, the follicular phase, ovulation, and the luteal phase. The highest value is seen during ovulation, with the exception of plasma testosterone levels (Table A2).

**Table 1:** Hormone concentrations (ng/mL) in plasma and urine for the phases of hormonal contraception (HC) and the natural menstrual cycle (MC).

Outcome	T (urine)	E (urine)	T/E (urine)	T (plasma)	A (plasma)
HC total (ng/mL)	1.78 ± 0.93	1.10 ± 0.54	1.70 ± 0.60	0.20 ± 0.06	0.36 ± 0.13
MC total (ng/mL)	5.91 ± 3.11	12.55 ± 9.33	0.60 ± 0.28	0.32 ± 0.07	1.08 ± 0.25
HC in (%)^a^	30.1	8.8	283.3	62.5	33.3

*Note:* Values are shown in mean ± standard deviation.

Abbreviations: A, androstenedione; E, epitestosterone; T, testosterone; T/E, testosterone/epitestosterone ratio.

^a^Related to MC.

Furthermore, urinary epitestosterone concentrations were found to be significantly elevated during the natural MC, particularly during ovulation and, to some extent, during the luteal phase, compared to the use of HC, resulting in a substantially lower urinary testosterone/epitestosterone (T/E) ratio during the MC.

## 4. Discussion

This single‐case study provides a comprehensive analysis of the intraindividual variability in endogenous anabolic steroid hormones in a female recreational athlete, contrasting HC use with natural MC phases. The findings reveal distinct differences in hormonal profiles, underscoring the impact of HC on physiological processes that should be considered in female athletes. The results indicate that androgen levels are, on average, lower in HC compared to MC. The range of HC concentrations is from 30.1% to 62.5% of the corresponding MC values, with the lowest relative proportion being observed for testosterone in urine (30.1%).

The hormonal suppression observed during HC use is consistent with its primary function of preventing ovulation and stabilizing androgenic activity [24]. This finding alligns with the findings of previous research, which has indicated that hormonal contraceptives can significantly alter endogenous hormone levels, particularly testosterone and androstenedione, leading to more stable but lower hormonal concentrations compared to the natural fluctuations seen in the MC [24, 25]. The lack of clear differences between the hormone‐free interval and the active contraceptive use is likely indicative of sustained continued suppression of endogenous steroid production. For instance, the lower urinary androgen levels observed during HC use may indicate a decrease in overall androgen synthesis and excretion. Testosterone is the primary bioactive androgen involved in the regulation of reproductive function, muscle growth, and bone health. Androstenedione and dehydroepiandrosterone, on the other hand, serve as circulating androgen precursors that contribute indirectly to androgenic activity through peripheral conversion to testosterone and other active androgens [26]. This suppression may have broader implications for athletic performance, as lower and stable hormone levels could potentially limit the physiological adaptations that typically occur with higher androgen content [27, 28] or in response to the cyclical nature of the MC [25]. The cyclical hormonal fluctuations observed during the natural MC, particularly the pronounced peak in testosterone and androstenedione around ovulation, suggest a physiological readiness for reproduction that may also enhance athletic performance and/or training adaptations. This finding aligns with the observations reported by Schulze et al. [30], who documented significantly elevated urinary concentrations of multiple steroid metabolites, notably epitestosterone, during the ovulation phase compared to the follicular phase. Consistent with previous reports, urinary epitestosterone concentrations in the present study also increased around ovulation and remained elevated during the luteal phase, which may reflect the known association between epitestosterone excretion and fluctuations in ovarian hormones, particularly estradiol [29]. Schulze et al. [30] identified epitestosterone as the urinary steroid most strongly affected by menstrual phase. In the present case study, urinary epitestosterone concentrations were found to be substantially higher during the natural MC than during hormonal contraceptive use. This resulted in a markedly reduced urinary T/E ratio under natural cycle conditions. This observation is consistent with the findings of previous studies, which have demonstrated that levels of epitestosterone excretion vary across the MC and are suppressed under the use of hormonal contraceptives. This is indicative of altered gonadotropin‐driven steroidogenesis and hepatic metabolism [15, 18, 30]. It has been demonstrated by earlier research that cycle‐based training programmes have the capacity to enhance performance by synchronizing training loads with hormonal fluctuations [31, 32]. The current findings of this study, which demonstrate elevated androgen levels during the ovulatory phase, lend support to the hypothesis that female athletes may benefit from tailored training programs that take these hormonal dynamics and their effects on perceptual responses into account [12]. Conversely, a study examining the impact of the MC on acute muscle protein synthesis revealed no significant differences between phases [33]. The consequences of these fluctuations at different physiological levels are therefore not yet fully understood.

Given the magnitude of variation and the single‐case design, observed changes in serum testosterone cannot be unequivocally attributed to MC phase. The observation of a peri‐ovulatory increase in androstenedione may be suggestive of phase‐dependent changes in precursor availability. However, it should be noted that this increase does not necessarily correspond with an increase in testosterone. This finding is consistent with the distinct regulation of the regulatory profiles of these steroids [20].

The urinary steroid profile obtained through GC‐MS/MS provides valuable insight into androgen metabolism, although its interpretation requires careful distinction between biologically active and inactive metabolites. Urinary androgen metabolites have been shown to reflect systemic metabolism and excretion to a greater extent than direct androgenic activity at the tissue level. Testosterone is the principal active androgen, while 5α‐androstanedione and 5α‐androstanediol represent precursors and downstream metabolites within the 5α‐reductase pathway. These substances reflect the capacity for 5α‐dihydrotestosterone formation and thus stronger androgenic signaling [34, 35]. Conversely, metabolites formed via the 5β‐pathway, such as 5β‐androstanedione, 5β‐androstanediol, and etiocholanolone, are devoid of androgen receptor activity and represent inactivation routes [20]. Androsterone, the primary 5α‐reduced end product, demonstrates only negligible androgenic potency yet functions as a reliable urinary marker of 5α‐reductase activity [36]. The relative abundance of 5α‐ compared to 5β‐metabolites, such as the androsterone/etiocholanolone ratio, is therefore commonly utilized to assess androgen metabolism in applied settings, including those in the fields of applied endocrinology and sports medicine [37]. While the sum of urinary androgens has been shown to correlate with circulating testosterone and thus provide evidence of androgen exposure [38], it is important that urinary androgen profiles provide complementary information on systemic steroid metabolism and excretion [36].

Furthermore, androgen secretion can be measured in different biological matrices. Plasma levels reflect immediate hormonal status, while urinary excretion provides a more stable, long‐term insight into androgen metabolism [20]. Urinary androgen metabolites have been demonstrated to reflect cumulative androgen metabolism and excretion and thus may offer a superior representation of overall androgen exposure. Conversely, circulating androgen concentrations in blood are more strongly influenced by acute physiological factors. This distinction assumes particular relevance when interpreting the findings of the present study, wherein fluctuations in plasma testosterone levels were observed that did not correspond with the more stable urinary testosterone levels. As Schiffer et al. [20] emphasize, the urinary metabolome reflects not only the synthesis of steroids but also their metabolic pathways and excretion processes. These processes can be influenced by various physiological factors, including HC use. This finding is in accordance with the results obtained by Mullen et al. [39], who observed considerable effects of emergency contraceptives on urinary steroid profiles. It has previously been demonstrated that the variability of steroid concentrations within an individual, as observed in urine, is significantly greater in comparison to that observed in serum, particularly in physically active women under hormonal contraceptive use. This finding underscores the utility of blood as a supplementary matrix for longitudinal hormone assessment [18].

Interestingly, the stable hormonal environment provided by HC may not only reduce endogenous hormonal fluctuations but could also influence other androgen‐dependent processes, such as mood and metabolic function. Research has indicated that hormonal contraceptives can lead to mood changes and weight gain, which may indirectly affect athletic performance [40]. The current study’s participant reported no significant adverse effects during the study, but the broader implications of HC use on mood and metabolic health warrant further investigation, particularly in high‐performance athletes [40]. The data also show that although plasma testosterone levels fluctuated during MC, they remained relatively lower and more stable with HC use [40].

This study has several strengths and limitations that should be considered. A significant strength of the study is the meticulous assessment of ovulation using continuous intravaginal core body temperature measurement, allowing precise cycle phase allocation. However, it should be noted that the present findings are based on a single recreational athlete and therefore may not be directly transferable to elite athletes with different training loads, recovery demands, and physiological stress. Furthermore, the concentrations of blood and urinary steroids are influenced by multiple confounding factors, including nutritional status (for example, whether the subject is fasted or fed), alcohol consumption, circadian rhythm, physical activity, and lifestyle‐related influences. Despite the longitudinal collection of samples and the implementation of a consistent time window for collection on a morning basis with the objective of reducing diurnal variability, these factors could not be fully controlled within the real‐world observational design. A longitudinal design encompassing a minimum of three cycles, in conjunction with the incorporation of additional markers such as sex hormone‐binding globulin or DHT, has the potential to offer a more comprehensive understanding of the subject. Recent studies have highlighted that, despite the growing interest in MC‐based training, there is still a paucity of robust evidence linking short‐term hormonal fluctuations to performance adaptations. This supports a cautious and descriptive interpretation of endocrine data [13]. Consequently, the results should be interpreted in a descriptive manner and with caution, particularly within a clinical context.

## 5. Conclusions

This single‐case study demonstrates intraindividual differences in urinary and plasma androgen profiles between hormonal contraceptive use and a natural MC. While it is not possible to draw causal or performance‐related conclusions from the findings, they provide descriptive insight into hormonal patterns that may inform future, larger‐scale investigations.

**Nomenclature**
FSH: Follicle‐stimulating hormone
HC: Hormonal contraceptive
LH: Luteinizing hormone
MC: Menstrual cycle.

## Author Contributions

Jana Nolte collected, analyzed, and interpreted the data and was a major contributor to writing the manuscript (original draft). Annekathrin Martina Keiler performed the hormone analysis. Kirsten Legerlotz was major contributor in writing, reviewing and editing the manuscript. Petra Platen, Annekathrin Martina Keiler, and Sven C. Voss were included in the conceptualization, methodology, supervision and writing, review, and editing of the manuscript.

## Funding

Open Access funding enabled and organized by Projekt DEAL.

## Disclosure

All authors have read and agreed to the published version of the manuscript.

## Ethics Statement

The participant gave written informed consent to the experimental procedures, which were approved by the institutional ethics committee of the Faculty of Sport Science at Ruhr University Bochum (EKS V 06/2022) and performed in accordance with the standards of ethics outlined in the Declaration of Helsinki.

## Consent

Informed consent was obtained from the participant involved in the study.

## Conflicts of Interest

The authors declare no conflicts of interest.

## Supporting Information

Additional supporting information can be found online in the Supporting Information section.

## Supporting information


**Supporting Information** Table A2: Raw data.

## Data Availability

The datasets used and/or analyzed during the current study are available as Supporting Information.
