# Supplementary material for: Intraindividual Comparison of the Anabolic Steroid Hormone Profile Between Hormonal Contraceptive Use and the Phases of a Natural Menstrual Cycle in a Recreational Athlete: A Single‐Case Observational Study
Source: Case Rep Endocrinol. 2026 Apr 8;2026:9648747. doi: 10.1155/crie/9648747 (PMC13059673; doi:10.1155/crie/9648747)
Supplement: Supplementary file 1 — Supporting Information Table A2: Raw data. [file CRIE-2026-9648747-s001.pdf]

Supplementary Material

2 Supplementary Data

Supplementary Table A2. Raw data

ID	Day	Phase	Androstenedione ng/mL	Testosterone ng/mL	17ß-Hydroxyprogesterone ng/mL	Progesterone ng/mL	Androsterone ng/mL	Etiocholanolone ng/mL	5a-androstanediol ng/mL	b-androstanedi ng/mL	Testosteron ng/mL	Epitestosteron ng/mL	T/E	Alcohol intake documentation	EtG 1 µg/mL
1.1	1	No HC					700,83	736,18	4,91	16,63	1,76	1,41	1,25	No	not measured
1.2	2	No HC	0,29	0,16	0,04		592,21	789,66	5,51	26,19	1,94	1,49	1,31	No	not measured
1.3	3	No HC					643,90	828,06	7,35	38,86	2,97	1,86	1,60	No	not measured
1.4	4	No HC	0,43	0,27	0,11		455,37	530,21	10,91	44,17	4,37	1,51	2,89	No	not measured
1.5	5	Ring intake					600,47	786,81	8,21	41,93	2,97	1,69	1,76	No	not measured
1.6	6		0,56	0,30	0,39		774,99	1092,16	7,45	35,98	2,28	2,74	0,83	No	not measured
1.7	7						564,01	831,22	4,78	21,09	1,49	1,71	0,87	No	not measured
1.8	8		0,34	0,11	0,06		807,31	1027,42	5,74	28,06	2,18	1,95	1,12	No	not measured
1.9	9						892,34	1017,77	6,52	39,38	2,47	1,35	1,83	No	not measured
1.10	10		0,33	0,24	0,12		419,08	510,98	2,80	13,63	1,16	0,67	1,73	No	not measured
1.11	11						453,44	526,25	2,87	13,92	1,25	0,60	2,10	No	not measured
1.12	12		0,30	0,17	0,10		319,57	297,48	5,26	22,16	1,70	0,70	2,42	No	not measured
1.13	13						436,10	427,36	2,47	12,02	0,99	1,34	0,74	No	not measured
1.14	14		0,28	0,17	0,10		335,67	329,19	2,26	9,55	0,86	0,56	1,55	No	not measured
1.15	15						277,40	242,56	1,69	7,32	0,66	0,47	1,39	No	not measured
1.16	16		0,28	0,18	0,11		421,06	392,18	2,17	11,24	1,02	0,66	1,55	No	not measured
1.17	17						428,16	395,06	2,74	10,02	0,89	0,51	1,73	No	not measured
1.18	18		0,23	0,15	0,06		705,18	486,35	5,40	19,26	2,10	1,10	1,90	No	not measured
1.19	19						227,27	182,09	1,12	5,09	0,96	0,64	1,49	No	not measured
1.20	20		0,67	0,25	0,60		593,79	484,73	4,03	10,82	1,37	1,00	1,37	No	not measured
1.21	21						250,52	218,30	1,40	4,46	0,60	0,39	1,54	No	not measured
1.22	22		0,31	0,14	0,11		555,56	439,22	3,29	9,01	1,25	0,69	1,82	No	not measured
1.23	23						996,82	834,81	5,30	16,13	1,91	1,28	1,49	No	not measured
1.24	24		0,26	0,20	0,07		495,90	401,80	4,03	14,33	1,69	0,86	1,96	No	not measured
1.25	25						363,38	288,33	1,71	6,49	0,86	0,64	1,35	Yes	not measured
1.26	26	Ring removal	0,48	0,24	0,10		213,97	158,97	9,35	30,99	3,75	1,04	3,60	No	not measured
1.27	27	No HC					758,49	637,68	5,18	22,55	2,36	1,06	2,22	No	not measured
1.28	28	No HC	0,26	0,14	0,09		753,79	688,89	5,73	18,68	2,07	0,95	2,18	No	not measured
	1	M												No	
	2	M												No	
2,1	3	M	0,89	0,44	N/A	N/A	1401,71	1549,38	13,49	48,77	4,75	5,75	0,83	No	
	4	M												No	
2,2	5	M	0,94	0,32	N/A	N/A	609,84	835,25	8,86	27,76	2,56	3,54	0,72	No	
	6	FP												No	
2,3	7	FP	1,07	0,3	N/A	N/A	717,65	818,67	13,52	34,10	3,65	4,01	0,91	No	
	8	FP												No	
	9	FP												No	
2,4	10	FP	0,79	0,26	N/A	N/A	1169,45	1351,16	15,47	46,77	5,57	7,13	0,78	No	
	11	FP												No	
2,5	12	FP	1,35	0,35	N/A	N/A	1781,43	2198,38	32,86	96,99	9,82	12,47	0,79	No	20
	13	FP												No	
2,6	14	Ovulation	1,56	0,27	1,95	1,21	2034,89	2237,94	33,18	86,49	12,20	35,24	0,35	No	20
	15	LP												No	
	16	LP												No	
2,7	17	LP	0,7	0,26	1,04	6,7	1066,31	1008,27	18,12	42,16	5,75	20,55	0,28	No	
	18	LP												No	
2,8	19	LP	1,1	0,37	3,95	23,88	664,05	743,94	9,56	32,44	2,93	11,11	0,26	No	
	20	LP												No	
2,9	21	LP	1,19	0,23	2,99	20,04	1406,09	1506,74	17,55	72,61	6,48	22,58	0,29	No	
	22	LP												No	
	23	LP												No	
2,1	24	LP	1,19	0,41	1,37	10,15	809,17	916,29	9,02	31,33	2,92	7,58	0,39	No	
	25	LP												No	
2,11	26	LP	1,14	0,26	N/A	1,78	1876,96	2062,27	25,14	75,74	8,34	8,03	1,04	No	

Alcohol intake:

During the natural menstrual cycle, low amounts of alcohol were found on two isolated days without any alcohol documentation of the participant, resulting in very low urinary EtG concentrations (20 µg/mL). These low EtG values were not associated with any discernible alterations in urinary testosterone, epitestosterone, the T/E ratio, or serum steroid concentrations. Although some physiological variability ("background noise") of the urinary T/E ratio was observed across the cycle, this variability was not systematically linked to alcohol intake. The highest T/E ratio observed during the natural cycle occurred on one of the days following alcohol intake. However, a comparably high T/E value was also observed during menstruation in the absence of alcohol consumption, suggesting that this variation reflects normal intraindividual variability rather than a specific ethanol effect. In contrast, during the hormonal contraceptive phase, higher alcohol intake was self-reported without confirmation of elevated urinary EtG concentrations (not measured). This coincided with a distinct deviation in the urinary steroid profile in the sample collected on the subsequent day, indicating a measurable alcohol-related effect on steroid metabolism under hormonal contraceptive conditions. This time point occurred during the hormone-free interval of the intravaginal ring cycle, a phase that is known to be associated with transient increases in endogenous steroid secretion. The observed increase in urinary testosterone and T/E ratio is therefore most likely attributable to the combined influence of acute alcohol intake and the hormone-free phase, rather than representing a generalizable effect of hormonal contraception.
